# A Prosthodontic Treatment Plan for a Saxophone Player: A Conceptual Approach

**DOI:** 10.3390/dj6030033

**Published:** 2018-07-18

**Authors:** Miguel Clemente, Joaquim Mendes, André Moreira, Afonso Pinhão Ferreira, José Manuel Amarante

**Affiliations:** 1 Faculty of Dental Medicine, University of Porto, 4200-393 Porto, Portugal; miguelpaisclemente@hotmail.com (M.C.); andre.luis.sa.moreira@gmail.com (A.M.); 2 Department of Mechanics, Faculty of Engineering, University of Porto, 4200-465 Porto, Portugal; 3 Department of Orthodontics, Faculty of Dental Medicine, Department; University of Porto, 4200-393 Porto, Portugal; aferreira@fmd.up.pt; 4 Department of Surgery, Faculty of Medicine, University of Porto, 4200-319 Porto, Portugal; amarante@med.up.pt

**Keywords:** oral rehabilitation, piezoresistive sensors, prosthodontics, embouchure

## Abstract

Introduction: A wind instrumentalist was diagnosed with a periapical lesion on tooth 21. The prosthetic rehabilitation options were considered with respect to the embouchure mechanism of the saxophonist. The underlying mechanism associated with the embouchure of the saxophone player was observed in this particular case in order to understand if asymmetrical forces were transmitted to the upper central incisors. Periapical lesions can be harmful to the oral health of musicians. The treatment options thus have to be taken into consideration with special focus on the need for oral rehabilitation on the anterior maxilla. Material and Methods: The patient underwent a radiographic examination with a panoramic X-ray. Subsequently, two piezoresistive sensors (FlexiForce™) were placed on the upper surface of the mouthpiece in order to quantify the pressure applied to the central incisors during the embouchure. In order to understand the values involved during this procedure, the saxophone player was required to play three different notes at different pitches: high, medium, and low. This procedure was repeated three times for each pitch in order to obtain a medium value for each note. Signal acquisition was obtained within software developed for this purpose, with the voltage output observed in LabView 2011^®^. Results: The panoramic X-ray showed a periapical lesion with the characteristics of a radicular cyst on tooth 21. The FlexiForce™ piezoresistive sensors allowed us to find that greater force (kg) was being applied to tooth 11 in comparison to tooth 21 during the embouchure mechanism. Conclusions: The sensors used in this research are acceptable for identifying the tooth where the greatest pressure is applied during the mouthpiece stabilization. In the case of executing an oral rehabilitation procedure for wind instrumentalists, a clinical examination can be complemented with the aid of bioengineering and the inherent development of sensor technology in order to better understand the embouchure mechanism. Likewise, the prosthetic rehabilitation should be taken into consideration in order to provide minimal changes to the musician’s performance.

## 1. Introduction

Wind instrumentalists display complex neuromuscular activity during their musical performances. As such, the embouchure and orofacial structures should be of major interest for dental practitioners. The orbicular muscles of single-reed instrument players can achieve bioelectric potential values of 40 µV, which may correspond to the double the normal values associated with the mastication process [1]. In fact it is of general knowledge that the main masticatory functions are mastication, deglutition, and speech, whereas other activities, such as playing wind instruments, bruxism, or nail-biting, can be considered a parafunction [1,2]. The stomatognathic apparatus has to maintain the muscle activity present on the cranio-cervico-mandibular complex (CCMC) of the musician for many hours per day.

Sustained incisal pressure by the upper central incisors on the mouthpiece and the inherent habit of tolerating these forces is typical of wind instrumentalists. This force can be denominated as the maximal embouchure force (MEF), which according to the different registration tones (brass or treble) can induce different pressures on the orofacial tissues [3]. To what point the MEF can be maintained for long periods is a crucial aspect for wind instrumentalists and in this particular case, saxophonists. Saxophone players create an MEF during the parafunctional activity of playing a wind instrument, and this continuous activity can lead do the appearance of pain or sensitivity on the upper teeth or on the lower lip. This can happen on the lower lip since single-reed instrumentalists, such as clarinet and saxophone players, place the mouthpiece between the lower lip and the upper central incisors. The lower lip is folded backwards over the anterior central incisors, while the upper central incisors are placed against the upper part of the mouthpiece, promoting the grip of the embouchure. The impact of sustained pressure during the embouchure and musical performance is directly proportional to the physiological tolerance of each musician and is related to their orofacial features. The type of occlusion, as well as the crowding, inclination, and rotation of the anterior teeth, can determine the embouchure of a wind instrumentalist. These musicians can require a higher amplitude of mouth opening if they have a deep overbite and play a single-reed instrument like a clarinet. In addition, a major anterior movement of the mandible can be promoted in trumpet players with Class II, Div 1 malocclusions when playing in low pitch. However, wind instrument players unfortunately do not take orofacial considerations as a major issue of interest. From a clinical point of view, in dentistry these are emerging matters that single-reed, double-reed, and brass instrumentalists should take into account in their daily practice [4].

The inclination of the mouthpiece inside the mouth can differ between clarinet and saxophone players. While the clarinet has an insertion of the mouthpiece more parallel to the inclination of the upper central incisors, the saxophone is placed in a more horizontal position. With respect to the grip of the embouchure, this can also be determined by the incisal edge of the upper central incisors, where any kind of irregularity or alteration on its inclined plane or in its smoothness will induce the adoption of a different position of the mouthpiece with respect to the upper teeth. With all these particularities, can we say there is a perfect embouchure? From a functional point of view the answer is yes, because the interface of the teeth and the mouthpiece will be adapted within the individual anatomical characteristics of each saxophonist.

The masticatory neck and trunk muscle activity have an interrelation with the occlusion of the musician and his/her embouchure. Temporomandibular disorders (TMD; where myofascial pain/dysfunction of the masticatory and cervical muscles can be present) along with internal derangement of the temporomandibular joint (TMJ; which can be detrimental for asymmetrical embouchures) can be expected [2,4,5]. As mentioned previously, wind instrumentalists usually tend to manage and adapt themselves in order to achieve a perfect embouchure during their musical performance. In order to investigate to what extent there are asymmetrical embouchures in saxophone players we should observe the saxophone player performing his or her embouchure during the dental appointment. Likewise, a complementary radiological examination should be done in order to observe the orofacial structures of the musician.

The present study aimed to evaluate the pattern of the embouchure of a saxophone player who attended a dental appointment for a routine consultation, referring specifically that tooth 21 had an abnormal colour. This professional wind instrumentalist wanted to understand the possible treatment options and the following rehabilitation procedures in order eliminate the radicular cyst. In this particular case we must consider the enormous importance of tooth 21 with respect to the musical career of the patient, considering the professional, social, psychological, and economic impacts of treatment. A single missing anterior tooth is a challenge for most clinicians to restore in terms of aesthetics and function. Due to the multiple treatment modalities that are available to replace a single missing tooth, there are always different advantages and disadvantages for each case [6]. Patient financial status, gender, age, public awareness, knowledge, pain, and dental phobia are important factors and may affect the patient’s decision to receive a treatment or simply refuse it altogether [7,8]. To decide with the patient the best prosthodontic option to replace a single tooth or multiple teeth, important factors such as smoking habits, presence and state of periodontal disease, oral plaque, parafunctional habits, failure, complications, and disadvantages should be taken into account. Pre-treatment information is of great importance during the prosthodontic rehabilitation and maintenance phase [9]. Female patients tend to prefer fixed implant treatment as option as compared to a removable treatment modality, suggesting that females are more apprehensive about their appearance. Education is another important factor that might increase the patient’s awareness with respect to the importance of tooth replacement [10].

## 2. Materials and Methods

**Figure 1:**
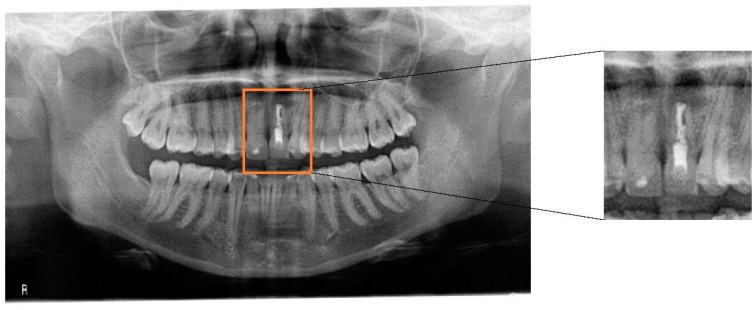
Panoramic radiograph of the patient. A radiolucid periapical lesion on tooth 21 can be observed, which did not disappear after endodontic treatment and apicoectomy.

**Figure 2:**
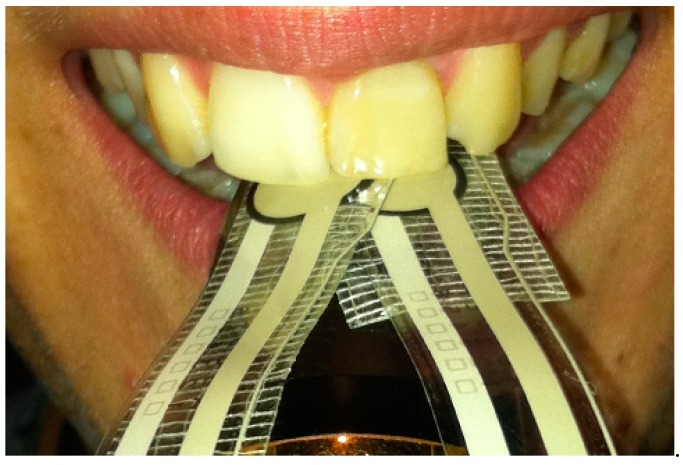
Extra-oral photograph of the embouchure mechanism and the piezoelectric sensors positioned in relation to the upper central incisors.

This case report is of a female professional saxophone player. The collection of information on the patient’s general health was performed prior to the clinical examination. The musician underwent a radiographic examination which consisted of the acquisition of a panoramic X-ray (Figure 1). Subsequently, two piezoresistive sensors (FlexiForce™, Tekscan, Boston, MA, USA, 0.07 kgf/cm^2^) were placed on the upper surface of the mouthpiece in order to quantify the pressure applied to the central incisors during the embouchure. In order to understand the values involved during this procedure, it was requested that the saxophone player perform three different notes at different pitches: high, medium, and low. This procedure was repeated three times for each pitch in order to obtain a medium value for each note. The sensors were previously calibrated and integrated with the recommended circuit from Tekscan and connected to a four-channel Universal Analog Input Module 9219 mounted in a NI-USB 9162 Carrier (National Instruments, Austin, TX, USA). To achieve the recommended circuit, a TL074 STMicroelectonics operational amplifier was used. Sensor characterization was made through polynomial regression. In this way, four different weights (100 g, 250 g, 500 g and 1000 g) were applied to the sensors and the voltage output was observed with a LabVIEW 2011 Carrier (National instruments, Austin, TX, USA). The FlexiForce Model A201-1 lb/sqi “TekScan” sensors were applied at specific locations on the mouthpiece as seen in Figure 2, where there is the usual tooth pressure.

## 3. Results

The values were registered when recording the force applied to the musician’s teeth (11 and 21), while playing the saxophone. As seen in Table 1, there are a wide range of values when comparing the force applied between teeth 11 and 21. We can observe greater values of pressure registered by the sensor of tooth 11 in both mean and maximum values. The musician applied greater force and pressure to the teeth during the lower-pitched notes, especially to tooth 11.

## 4. Discussion

This paper intends to approach a theoretical and conceptual presentation where piezoelectric sensors can be a complementary tool in the assessment method for the diagnosis of embouchure force measurement. Currently, this method can be considered the most accurate exam for identifying the central incisor that applies the most pressure during musical performance [4]. In this particular case, the authors described the application of the piezoresistive sensor as the gold standard for this purpose in order to study the existence of an asymmetrical embouchure. During this research, the data obtained with respect to the wind instrument embouchure are the maximum and average values. The authors intended to quantify and present the results for the mean and maximum values because the mean represents the forces that are being transmitted by the mouthpiece to the teeth during most of the musical performance, while the maximum values correspond to the greatest pressure that is produced during the embouchure, which could eventually be harmful for the orofacial structures. A possible explanation for the difference in force found between teeth 21 and 11 may be the fact that unconsciously the musician rotates her mouthpiece to her right upper central incisor, protecting the left upper central incisor which has a periapical lesion. Another theory is that the musician has a higher proprioception of the embouchure when stabilizing the saxophone’s mouthpiece on her right upper central incisor. In a regular saxophonist, similar values would be expected in both upper central incisors. Relative to the values and the pitch, saxophonists normally apply a greater force and pressure to the teeth when playing higher-pitched notes. However, the musician in this case showed the opposite—greater values in lower-pitched notes. Furthermore, it could be said that the loss of vitality and the periapical lesion found in tooth 21 may have changed the embouchure mechanism of the musician in question.

The capacity to play a wind instrument is dependent upon the balance of the CCMC and its healthy state. This single-reed instrument player presented an inflammation at the periapical region of the tooth, which eventually developed into granulation tissue from the wounding afforded by pulpal inflammation. This inflamed granulation tissue has been referred to historically as a periapical granuloma. Radiographically, the lesion is well-marked by a radiolucency that is present and associated with the apex of the tooth. Normally, a presumptive clinical-radiographic impression of a periapical cyst can be made in cases where the radiolucency has a diameter of greater than 20 mm [11]. Radicular cysts are common lesions in daily dentistry practice. Treatment is conventional root canal therapy or enucleation of the cyst/granuloma, often at the time of extraction or during an apicoectomy [12,13]. Usually endodontic re-treatment resolves this type of periapical lesions by abundantly irrigating with sodium hypochlorite and with a more condensed root canal filling. The success of the re-retreatment relies on reducing the number of microorganisms at the bone level and allowing a correct healing response [14,15].

According to the prosthetic rehabilitation of the single tooth of this saxophone player, there are different treatment options. The systematic review conducted by Edelmayer et al. showed that in most studies, dental implants and fixed dental prosthesis (FDPs) are preferred, as compared to removable partial dentures (RPDs) as an alternative for missing single teeth [9]. The authors of the previous study found that the high cost was the main reason for refusal of treatment options; on the other hand, one-third of the patients were afraid of the treatment or feared possible risks and side effects, and a small portion of the sample criticized the long duration of the treatment [9]. In cases of single tooth replacement, a removable partial denture has a number of disadvantages and therefore the cases may be limited. Usually an acrylic denture is used to improve the patient’s aesthetics, but normally the patient does not desire or accept a removable partial prosthesis as a substitute for a single missing tooth, especially an anterior tooth [16]. In this particular case, the saxophonist had already been treated with an endodontic procedure and an apicoectomy on tooth 21. With the extension of the existing cyst, a possible solution could be tooth extraction and the placement of a removable prosthesis over a six-month time frame during the bone healing process in order to place an implant.

Nevertheless, a removable prosthesis would in theory create difficulties in the stabilization of the mouthpiece during the musical performance. Hypothetically, this would happen if there was an equilibrium of the applied pressures induced by the central incisors on the mouthpiece, but what our study supports is that there is a higher demand placed on tooth 11 during the embouchure mechanism. Thus, a removable prosthesis on tooth 21 would not make a huge difference to the stabilization of the mouthpiece since the pressure applied on this tooth had a mean of 8 g compared to 72 g on tooth 11.

As an alternative, a fixed partial denture from tooth 11 to 22 could be an option, especially considering the reduced time and cost for this procedure. However, in addition to the inherent cost of the treatment, the damage of the neighbouring teeth for crown preparation is also of importance. Moreover, this solution would place higher demand on the adaptation of the musician’s embouchure, since we would be changing not only the neuromuscular pattern of the embouchure (with a different distribution of pressure induced on the mouthpiece), but also the biomimetics of tooth 11.

The implant procedure would avoid tooth preparation, which sometimes requires additional endodontic treatment. On the other hand, the placement of a dental implant on the post-extraction socket of tooth 21 would not be recommended due to the existing chronic periapical infection, as this may be considered as a risk factor (but not an absolute contraindication) for immediate implant placement. However, debridement of the alveolus should be performed and the implant surgery scheduled six months later. More than the cost, time required, and difficulty in performing treatment, a long-term solution for the specific case of our patient should be opted for [10]. Haistreter and Jiang affirmed that dental implants can provide various clinical and quality of life advantages compared to FDPs and RPDs [17]. Dental implants are a viable solution for the restoration of single tooth gaps, with high survival and success rates in the short and long term [18,19]. Branemark in 1977 [20] introduced the osseointegration theory, which states that the healing period of an implant should be at least 3–4 months without loading to achieve osseointegration of the dental implant [21]. Moreover, micromotion would disturb the healing process by causing a fibrous scar tissue that would separate the implant from the bone instead of promoting bone apposition in the implant surface [22,23]. The following events of micromotion could in the end lead to the failure of the implant [23]. 

In this particular case, due to the pathologic process involved in the surrounding bone tissue of tooth 21, the matter of immediate loading should not be considered, nor early loading. For this purpose, conventional loading is the most adequate loading protocol of the three options in implantology. However, when assessing prosthetic rehabilitation options in professional wind musicians, careful selection and specific treatment protocols are required so that the musician may continue playing.

The conceptual approach explained in this article intends to highlight the possibility of using the piezoelectric sensors in the analysis of the clarinettist’s embouchure in order to understand and quantify the existing forces. In this case the wind instrument applies very low pressure on the tooth that will be replaced, and therefore the prosthondontic solution adopted will not change the embouchure of the musician since the greatest pressure is applied to tooth 11, which is not the target of the treatment. This theoretical analysis of the embouchure with the aid of piezoelectric sensors could be useful in the dentistry field. Nevertheless more studies should be made in order to provide greater knowledge on its practicability from a clinical point of view.

## 5. Conclusions

MEF measurements are a relatively new modality that can be very useful in dental practice as a complementary tool in the diagnosis of wind instrumentalists’ embouchures. This system can be used as a part of complex treatment, as the case of the prosthodontic rehabilitation of this saxophone player. Therefore, this study has shown the effectiveness of this method in the elaboration of a treatment plan. The field of dentistry is undergoing considerable transformation with respect to the orofacial issues of wind instrumentalists, specifically, the musicians’ embouchure.

Wind instrumentalists should regularly visit the dentist in order to evaluate their general oral health. Regarding the rehabilitation procedure, there should be a proposal to implement the most conservative treatment. If not, the recommended prosthetic rehabilitations options should embrace the physiological requirements of the wind instrumentalists’ embouchure while taking into account psychosocial aspects.

The professional activity of these kind of patients have to be taken in consideration since there are economic matters related to the inhibition of musical performance during the prosthetic rehabilitation procedures. The piezoresistive FlexiForce™ sensors provide valid information that quantifies the pressure induced by the upper central incisors on the mouthpiece. In the future, this technique can be implemented in the screening of wind instrumentalists embouchure before and after oral rehabilitation.

## 6. Patents

No patents may or will result from this work.

## Figures and Tables

**Table 1:** Table with the mean and maximum forces and pressures applied by the saxophone to tooth 11 (right upper central incisor) and 21 (left upper central incisor) with different pitches.

Pitch	Force (kgf)				Pressure (kgf/cm^2^)			
	Mean		Maximum		Mean		Maximum	
*Tooth*	*11*	*21*	*11*	*21*	*11*	*21*	*11*	*21*
High	0.038	0.009	0.096	0.018	0.014	0.003	0.034	0.007
Medium	0.070	0.005	0.185	0.016	0.025	0.002	0.066	0.006
Low	0.108	0.010	0.201	0.031	0.039	0.003	0.071	0.011
